# Insight into the Formation of Cocrystal and Salt of Tenoxicam from the Isomer and Conformation

**DOI:** 10.3390/pharmaceutics14091968

**Published:** 2022-09-19

**Authors:** Yifei Xie, Penghui Yuan, Tianyu Heng, Lida Du, Qi An, Baoxi Zhang, Li Zhang, Dezhi Yang, Guanhua Du, Yang Lu

**Affiliations:** 1 Beijing City Key Laboratory of Drug Target and Screening Research, National Center for Pharmaceutical Screening, Institute of Materia Medica, Chinese Academy of Medical Sciences and Peking Union Medical College, Beijing 100050, China; 2 Beijing City Key Laboratory of Polymorphic Drugs, Center of Pharmaceutical Polymorphs, Institute of Materia Medica, Chinese Academy of Medical Sciences and Peking Union Medical College, Beijing 100050, China; 3 Shandong Soteria Pharmaceutical Co., Ltd., Laiwu 271100, China; 4 Laboratory of Xinjiang Uygur Medical Research, Xinjiang Institute of Materia Medica, Urumqi 830004, China

**Keywords:** tenoxicam, cocrystal, salt, conformation, pKa, theoretical calculation

## Abstract

Tenoxicam (TNX) is a new non-steroidal anti-inflammatory drug that shows a superior anti-inflammatory effect and has the advantages of a long half-life period, a fast onset of action, a small dose, complete metabolism, and good tolerance. Some compounds often have tautomerism, and different tautomers exist in different crystalline forms. TNX is such a compound and has three tautomers. TNX always exists as the zwitterionic form in cocrystals. When the salt is formed, TNX exists in the enol form, which exhibits two conformations depending on whether a proton is gained or lost. Currently, the crystal structure of the keto form is not in the Cambridge Structural Database (CSD). Based on the analysis of existing crystal structures, we derived a simple rule for what form of TNX exists according to the pKa value of the cocrystal coformer (CCF) and carried out validation tests using three CCFs with different pKa values, including *p*-aminosalicylic acid (PAS), 3,5-dinitrobenzoic acid (DNB), and 2,6-dihydroxybenzoic acid (DHB). The molecular surface electrostatic potential (MEPS) was combined with the pKa rule to predict the interaction sites. Finally, two new cocrystals (TNX-PAS and TNX-DNB) and one salt (TNX-DHB) of TNX were obtained as expected. The differences between the cocrystals and salt were distinguished by X-ray diffraction, vibration spectra, thermal analysis, and dissolution measurements. To further understand the intermolecular interactions in these cocrystals and salt, the lattice energy and energy decomposition analysis (EDA) were used to explain them from the perspective of energy. The results suggest that the melting point of the CCF determines that of the cocrystal or salt, the solubility of the CCF itself plays an important role, and the improvement of the solubility after salt formation is not necessarily better than that of API or its cocrystals.

## 1. Introduction

**Figure 1:**
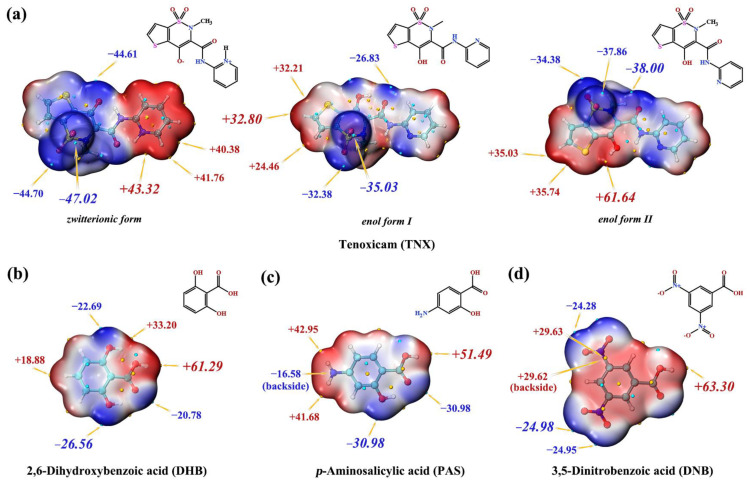
Molecular structures and MEPS of API (**a**) and CCFs (**b**–**d**).

Tenoxicam (TNX) is a non-steroidal anti-inflammatory drug developed by Roche in 1976 and first listed in Switzerland in March 1987 under the trade name Tihotil. It exerts pharmacological effects by inhibiting cyclooxygenase, reducing the synthesis of local prostaglandins, and inhibiting the chemotaxis of leukocytes and the release of lysosomal enzymes [1]. Its anti-inflammatory effect is superior to that of aspirin, mefenamic acid, and naproxen, and its efficacy is equal to that of piroxicam [2]. Its clinical dosage forms are tablets and suppositories [3], which have the advantages of a long half-life period (70~90 h), a fast onset of action, a small dose (20 mg/d), complete metabolism, hydrophilicity, and good tolerance. It is clinically used to treat arthritis, acute gout, frozen shoulder, etc. [4,5,6,7]. TNX is a Biopharmaceutics Classification System (BCS) class II drug that possesses a low solubility and high permeability [8]. Therefore, improving its solubility is an important way to improve bioavailability. The available methods include the formation of different polymorphs, salts/cocrystals, and other solid forms [9,10,11,12,13,14]. At present, there are five polymorphs and seven solvates of TNX that have been reported [15,16]. Conformation is an important factor leading to the polymorphism of TNX. There are three conformations of TNX: the enol form, keto form, and zwitterionic form, but TNX is mainly in the enol or zwitterionic form in known crystal structures (see Figure 1). The solubilities of different crystal forms are different according to Reference [17], and the orders are form II > form IV > form III > form I, and dioxane solvate > n, n-dimethylformamide solvate > acetonitrile solvate. Forms I and IV conform well with the keto form, while III conforms with the enol form in infrared spectra (IR), according to Reference [18]. However, a single-crystal X-ray diffraction analysis showed that form III and solvates of TNX exist in the zwitterionic form.

Pharmaceutical cocrystals and salts are both a kind of multicomponent drug system. Some physicochemical properties of API, such as solubility and thermal stability, can be significantly improved by forming cocrystals or salts with the proper coformer. Moreover, cocrystals and salts likely possess the advantage of multiple or synergistic pharmacological effects when the coformer has pharmacological activities. There are currently 11 cocrystals and salts reported in the CSD of the Cambridge Crystallographic Data Center (CCDC) database [19,20]. The main coformer types of salts or cocrystals are phenolic and benzoic acid compounds. All TNX exists in the zwitterionic form in cocrystals and in the enol form in salts, whereas there are two conformations, namely, enol form I and enol form II, in this work (see Figure 1). This is an interesting phenomenon, and Arkhipov et al. [21] analyzed why TNX was more likely to appear in the zwitterionic form rather than the keto or enol form in different crystal and cocrystal structures based on the role of the S bond. In this work, we obtained a similar result and further analyzed the reason from the perspective of the pKa of the coformer compound and the interaction energy. We derived a rough rule; that is, if the coformer compound is an acidic compound and its pKa value is less than 2, or the coformer compound is an alkaline compound and its pKa value is greater than 9, the salt will be formed in enol form I or enol form II, while with pKa values outside of this range, the cocrystal will usually be formed in the zwitterionic form. This simple rule helps explain why several phenolic compounds (pKa > 9) listed in Table 1 form cocrystals but not salt with TNX [22,23]. Detailed information on the crystals/salts of TNX is listed in Table 2.

Based on the above analysis, we selected *p*-aminosalicylic acid (PAS), 3,5-dinitrobenzoic acid (DNB), and 2,6-dihydroxybenzoic acid (DHB) as coformer compounds to prepare new cocrystals or salt to verify the rule. PAS possesses anti-inflammatory and antibacterial activity [24]. DNB also exhibits antibacterial activity [25]. They have great potential to improve or extend the pharmacological activity of TNX by forming a cocrystal or salt with the parent drug. DHB, as a phenolic compound, has poor biological performance [26], but it still possesses important meaning to verify the rule we derived. Moreover, all three coformers are commonly used coformers in pharmaceutical cocrystal and salt research due to their relatively high stability and solubility. The structural formulas of coformer compounds are shown in Figure 1. Two cocrystals and one salt were obtained by grinding under slurry conditions and using solvent evaporation methods as expected. The cocrystals and salt were characterized by single-crystal X-ray diffraction (SXRD), powder X-ray diffraction (PXRD), differential scanning calorimetry (DSC), infrared spectroscopy (IR), and Raman spectroscopy. In addition, theoretical calculations based on density functional theory (DFT) were used to predict the interaction sites using the molecular electrostatic potential surface (MEPS) and analyze the hydrogen bonding motifs and intermolecular interactions of cocrystals and salts, thus providing valuable insights for further cocrystal/salt research on TNX.

## 2. Materials and Methods

### 2.1. Materials

TNX (purity > 98%) was purchased from Xi’an Kechuang Pharmaceutical Co., Ltd. (Xi’an, China). PAS (purity > 98%), DNB (purity > 98%), and DHB (purity > 98%) were purchased from Hubei Wande Chemical Co., Ltd. (Wuhan, China). All analytical-grade solvents were purchased from Beijing Chemical Works (Beijing, China).

### 2.2. Methods

#### 2.2.1. Preparation

TNX-PAS and TNX-DNB Cocrystals and TNX-DHB salt were prepared for characterization and evaluation by grinding under slurry conditions [27,28,29,30] as follows: TNX (169 mg, 0.5 mol) and PAS (77 mg, 0.5 mol), DNB (106 mg, 0.5 mmol), and DHB (77 mg, 0.5 mmol) in a stoichiometric ratio of 1:1 were each weighed into a clean mortar and ground thoroughly with 1 mL of methanol for about 10 min, and then yellow powder samples were obtained.

#### 2.2.2. Crystallization

Crystals of the above cocrystals and salt for SXRD analysis were prepared by the slow solvent evaporation (SSE) method at room temperature as follows: TNX (169 mg, 0.5 mmol) mixed with PAS (77 mg, 0.5 mmol), DNB (106 mg, 0.5 mmol), or DHB (77 mg, 0.5 mmol) was dissolved in 30 mL of methanol and stirred for two hours. Then, the solutions were filtered and left to stand at room temperature for about two weeks, and flaked yellow crystals were obtained.

### 2.3. Powder X-ray Diffraction (PXRD) Analysis

PXRD experiments were performed on a Rigaku SmartLab 9 KW diffractometer with Cu Kα radiation (λ = 1.54178 Å) (Rigaku, Tokyo, Japan). The powder samples were scanned continuously with a coverage of 3–40° at a constant rate of 8°/min. Simulated PXRD patterns were calculated using Mercury software (v 4.1.0, Cambridge Crystallographic Data Center, Cambridge, UK) at a starting angle of 3°, a final angle of 40°, a step size of 0.02°, and a full width at half maximum of 0.15°.

### 2.4. Single-Crystal X-ray Diffraction (SXRD) Analysis

SXRD experiments were performed on a Rigaku MicroMax-002+ CCD diffractometer with Cu Kα radiation (λ = 1.54178 Å) (Rigaku, Americas, The Woodlands, TX, USA). Intensity data were collected at 293 K. Absorption correction and integration of the collected data were performed using the CrystalClear software package. Crystal structures were solved and refined through full-matrix least-square methods, which were performed using Olex2 and SHELXL crystallography software [31,32,33]. All non-hydrogen atoms were refined anisotropically. Hydrogen atoms linked to carbon atoms were fixed in geometrically constrained positions, whereas hydrogen atoms associated with nitrogen and oxygen atoms were located through the difference Fourier method [34,35].

### 2.5. Differential Scanning Calorimetry (DSC) Analysis

DSC thermograms were recorded with DSC 1 (Mettler Toledo, Greifensee, Switzerland) and STARe Evaluation software 16.0. Approximately 3–5 mg was weighed into an aluminum crucible and heated at a constant rate of 10 °C/min over a temperature range of 30−300 °C under atmospheric conditions.

### 2.6. Infrared Spectroscopy (IR) Analysis

IR experiments were performed on a Spectrμm 400 Fourier transform infrared spectrometer (PerkinElmer, Waltham, MA, USA). Experimental conditions included an attenuated total reflection accessory, a spectral scanning range of 4000–650 cm^−1^, a resolution of 4.000 cm^−1^, and a scan number of 16.

### 2.7. Raman Analysis

All FT-Raman spectra in this paper were recorded at room temperature in the range of 4000–100 cm^−1^ with a 3 s integration time using a Horiba HR Evolution FT-Raman spectrometer equipped with a 532 nm Nd and YAG laser beam for spectral acquisition.

### 2.8. Theoretical Computation

The B3LYP-D3/6-311G + (d, p) level was employed for all hydrogen atom geometry optimizations, while all heavy atoms were observed at the original X-ray coordinates, and the same level was used for frequency and single-energy calculations using the Gaussian 16 package [36] based on density functional theory (DFT). The lattice energy calculations were carried out on the cocrystals and salt using CRYSTAL17 software [37] at the B3LYP/6-31G (d, p) level based on single-crystal structures. The EDA of the intermolecular interactions of the cocrystals and salt was carried out based on the generalized Kohn–Sham EDA (GKS-EDA) method by using the XEDA program [38,39,40]. The Multiwfn 3.8 package was used for all wave function analyses [41].

### 2.9. Dissolution Measurements

Dissolution measurements were investigated by the basket method by using an RC12AD dissolution instrument (Tianjin Tianda Tianfa Technology Co., Ltd., Tianjin, China). According to a previous study [42], it is necessary that all of the particle sizes of TNX, cocrystals, and salt are at the same level when the same operation is performed on these samples. To do so, the powder samples of TNX, two cocrystals, and one salt were milled and sieved through 100-mesh sieves to minimize the size influence on the results. The temperature and rotation speed were set to 37 °C and 160 r·min^−1^. The dissolution mediums were water (pH 7.0), phosphate buffer (pH 6.8), acetate buffer (pH 4.5), and hydrochloric buffer (pH 1.2). Accurately weighed samples (containing 60 mg of TNX) were each added to 900 mL of dissolution mediums. The sampling points were at 5, 10, 15, 20, 30, 45, 60, 90, 120, 180, 240, 360, 480, 600, and 720 min. The concentrations of TNX were quantified on an Agilent 1200 Series HPLC system (Agilent Technologies, Santa Clara, USA) with a Sil-Green C_18_ HPLC column (4.6 mm × 250 mm, 5μm) and a UV detector at a wavelength of 257 nm. The mobile phase was methanol–0.04% formic acid aqueous solution (61:39). The flow rate and column temperature were set to 1 mL·min^−1^ and 25 °C.

## 3. Results and Discussion

### 3.1. Prediction of Interaction Sites

Based on the structural analysis of TNX cocrystals in CSD, TNX exists in the zwitterionic form in cocrystals. During salt formation, TNX can act as either an acid (proton donor) or a base (proton acceptor). As a base, the conformation of TNX is usually in the form of enol form I, and as an acid, it is usually in the form of enol form II. According to the results of previous studies [43,44,45], the MEPS of a compound with different conformations also shows differences, which will lead to different intermolecular interaction sites. Therefore, we calculated the MEPS of CCFs and different conformations of TNX and carried out a prediction analysis of the interaction sites combined with the pKa rule.

The pKa of TNX is 4.50, and the pKa values of CCFs, including DHB, PAS, and DNB, are listed in Table 1. According to the derived pKa rule, the pKa of PAS and DNB is 3.58 and 2.77, which indicates cocrystal formation because their pKa values are more than 2. The pKa of DHB is 1.30, which indicates salt formation because its pKa is less than 2, and TNX acts as a base.

The MEPS of the zwitterionic form of TNX showed that the electron-rich region was on the left side, and the electron-deficient region was on the right side. Due to electrostatic attraction, two TNX molecules tend to form a dimer side by side in opposite directions. Thus, due to the existence of a local minimum point (−44.61 kcal/mol) near the hydroxyl oxygen where proton transfer occurred, it can interact with the global maximum point existing in the MEPS of PAS (+51.49 kcal/mol) or DNB (+63.30 kcal/mol) molecules to form hydrogen bonds. Only one proton acceptor site exists at the MEPS of enol form I of TNX, i.e., the nitrogen of the pyridine ring. Although there are three proton donors in DHB, the hydroxyl proton in the carboxyl group is more active (+61.29 kcal/mol) and will be preferentially transferred. At the MEPS of enol form II of TNX, the hydroxyl proton on the thiazine ring is more active (+61.64 kcal/mol) and will be preferentially transferred. Due to proton transfer, the distribution of molecular electron density changes, and the MEPS also changes, so the possible action sites of the salt cannot be predicted according to the MEPS in Figure 1.

### 3.2. Single-Crystal X-ray Diffraction (SXRD) Analysis

The structural details of two cocrystals and one salt were revealed by X-ray crystallography. All crystallographic data were deposited in CSD with deposition numbers 2174285–2174287. The main crystallographic data of the three novel crystals are summarized in Table 2.

**Figure 2:**
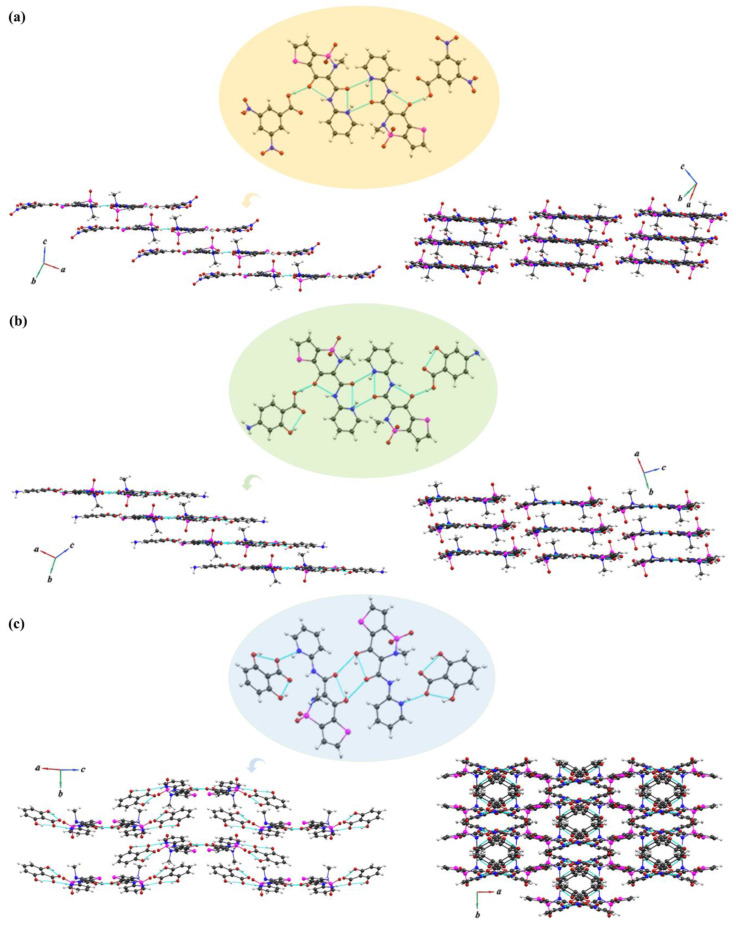
Hydrogen bond motifs and molecular stacking mode of (**a**) TNX-DNB, (**b**) TNX-PAS, and (**c**) TNX-DHB.

Based on SXRD analysis, the hydrogen bonding network in all crystals was systematically investigated. The hydrogen bonding parameters are listed in Table 3. The TNX-DNB cocrystal was crystallized in the triclinic space group $P\bar{1}$, and the asymmetric unit contained one TNX molecule and one DNB molecule. In this cocrystal, the TNX molecule also existed in zwitterionic form, having two intramolecular N-H…O hydrogen bonds (N_2_-H_2_…O_1_; N_3_-H_3_…O_4_). Two TNX molecules were linked by intermolecular N-H…O hydrogen bonds (N_3_-H_3_…O_4_) to form a centrosymmetric dimer with the $R_{2}^{2}\left(4\right)$ graph set notation. Two 3,5-dinitrobenzoic acid molecules were connected on each side through O-H…O hydrogen bonds (O_2D_-H_2D_…O_1_) to form a centrosymmetric tetramer. The tetramers were linked together through weak C-H…O interactions to form an approximately planar structure. The planes were linked by weak C-H…O interactions and S-S interactions to form a three-dimensional layered structure (Figure 2a). The TNX-PAS cocrystal was crystallized in the monoclinic space group *P*2_1_, and the asymmetric unit contained two TNX and two PAS molecules. The TNX molecule existed in the zwitterionic form in this cocrystal. There were two intramolecular N-H…O hydrogen bonds (N_2_-H_2_…O_1_ and N_3_-H_3_…O_4_) in the motif $S_{1}^{1}\left(6\right)$ in the TNX molecule. Two TNX molecules were connected by intermolecular N-H…O hydrogen bonds (N_3_−H_3_…O_8_; N_6_−H_6_…O_4_) to form a centrosymmetric dimer with the $R_{2}^{2}\left(4\right)$ graph set notation. Similar to the TNX-DNB cocrystal, in the TNX-PAS cocrystal, two TNX dimers linked two PAS molecules, respectively, through O-H…O (O_1A_-H_1A_…O_1_; O_4A_-H_4A_…O_5_) hydrogen bonds to form a tetramer. The tetramer extends infinitely on each side to form a planar structure through N-H…O (N_1A_-H_1AB_…O_3_; N_2A_-H_2AA_…O_7_) hydrogen bonds. Each plane was connected by O-H…O (O_3A_-H_3AA_…O_6_; O_6A_-H_6A_…O_2_) hydrogen bonds to form a three-dimensional layered structure (Figure 2b). The TNX-DHB salt was crystallized in the monoclinic space group *C*2/c, and the asymmetric unit contained one TNX molecule and one DNB molecule. Unlike the cocrystals, the TNX molecule existed as enol form I with intramolecular O-H…O hydrogen bonds (O_1_-H_1_…O_4_). Two TNX molecules were linked by intermolecular O-H…O hydrogen bonds (O_1_-H_1_…O_4_) to form a centrosymmetric dimer with the $R_{2}^{2}\left(4\right)$ graph set notation. Two DHB molecules were connected on each side through N-H…O hydrogen bonds (N_2_-H_2_…O_5_; N_3_-H_3A_…O_6_) to form a centrosymmetric tetramer. The tetramer formed a curved surface structure, and every two tetramers were interlaced face to face through weak C-H…O interactions. This structure extended indefinitely to form a three-dimensional network (Figure 2c). The result of SXRD analysis shows that the arrangements of cocrystals with the same TNX conformation were similar, but the arrangement of the salt was quite different from that of the cocrystals due to the change in the TNX conformation.

### 3.3. Powder X-ray Diffraction (PXRD) Analysis

**Figure 3:**
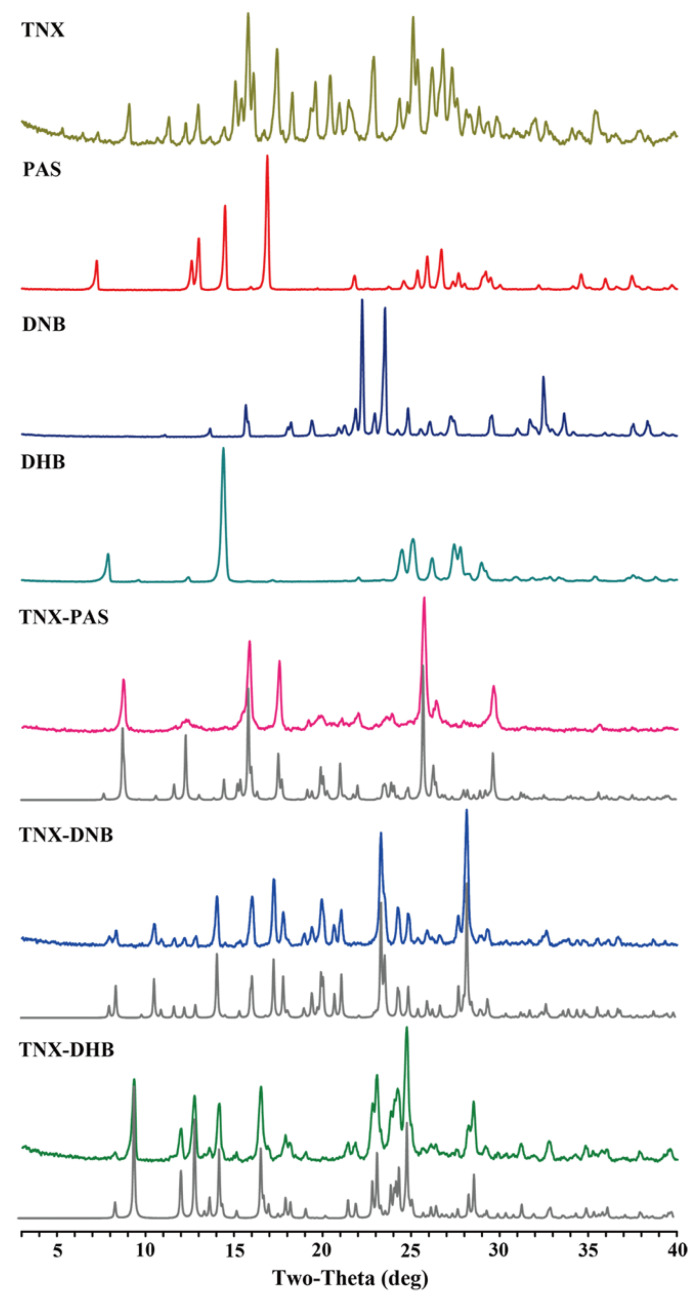
PXRD patterns of TNX, CCFs, and the corresponding cocrystals/salt.

The experimental PXRD patterns of the cocrystals and salt are shown in Figure 3, which differ from those of API and CCFs, indicating the formation of new phases. The simulated PXRD patterns colored in gray corresponding to the crystal structures were calculated using Mercury software. The experimental PXRD patterns of the cocrystals and salt agree well with the simulated ones. Their consistency indicates that the phase purities of the solid forms were good, and they can be used in other characterizations.

### 3.4. Infrared Spectroscopy (IR) Analysis

**Figure 4:**
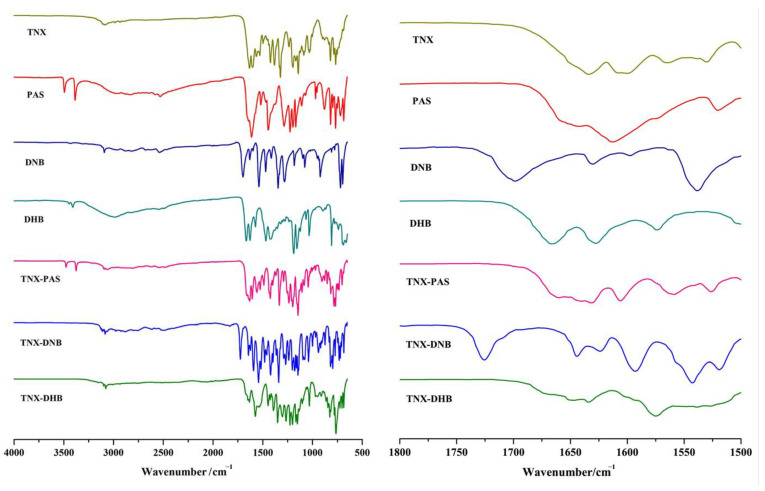
IR spectra of TNX, CCFs, and the corresponding cocrystals/salt (**left**); the region for OH stretch in IR spectra of TNX, CCFs, and the corresponding cocrystals/salt (**right**).

Vibrational spectroscopy is a reliable technique for characterizing hydrogen bonding and crystal packing in solids [46,47]. Changes in hydrogen bonding will lead to vibrational frequency shifts. The carbonyl stretching frequency of PAS (1611 cm^−1^), DNB (1698 cm^−1^), and DHB (1666 cm^−1^) was shifted to 1631 cm^−1^ in TNX-PAS, 1725 cm^−1^ in TNX-DNB, and 1646 cm^−1^ in TNX-DHB, revealing the influence of intermolecular O-H…O and N-H…O hydrogen bond formation, as shown in Figure 4 and Table 3.

### 3.5. Raman Analysis

**Figure 5:**
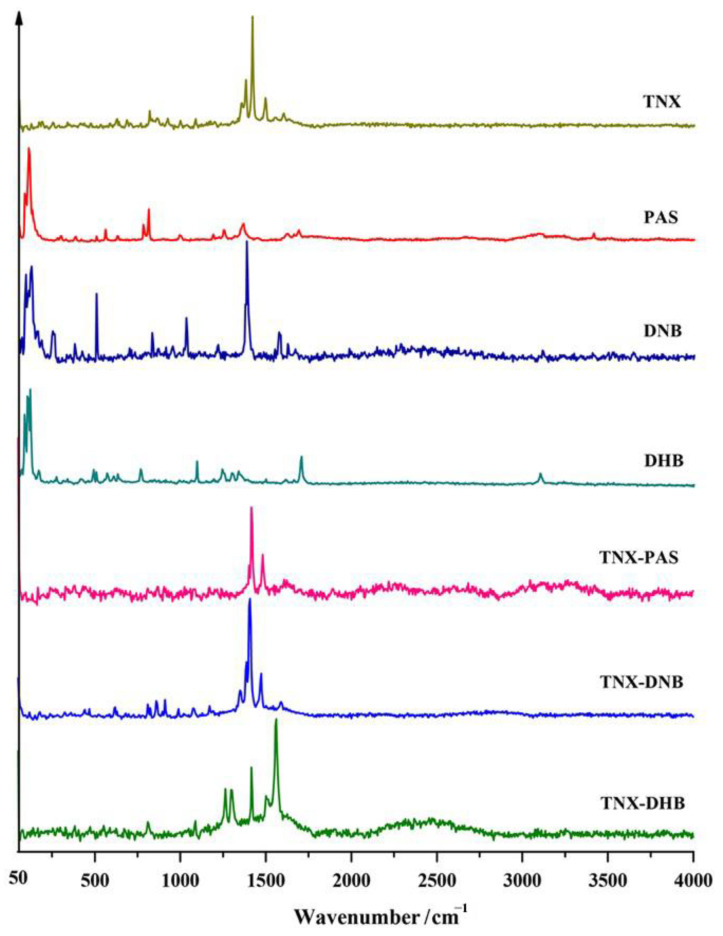
Raman spectra of TNX, CCFs, and the corresponding cocrystals/salt.

Raman spectroscopy is generated by changes in the polarization rate of molecules and is advantageous for identifying non-polar groups in molecules. This method can provide valid evidence to determine the form of tenoxicam and provide assistance in distinguishing between cocrystals and salts. Figure 5 shows the different Raman spectra between TNX, CCFs, and the corresponding cocrystals/salts. NH_2_-Ar exists in the PAS molecule, and there is a characteristic peak of ν_N-H_ at 3412 cm ^−1^, but no such peak appears in the spectrum of TNX-PAS, indicating that NH_2_ participates in eutectic formation. DNB and DHB are both derivatives of benzoic acid. There is a ν_C-H_ vibration of the benzene ring between 2800–3100 cm ^−1^ on the ring. The characteristic peaks at 3114 cm ^−1^ and 3101 cm ^−1^ in the Raman spectra of TNX-DNB and TNX-DHB disappear, indicating that there is π-π accumulation in the participating structure of the benzene ring. There is a strong peak of 1561 cm ^−1^ in TNX-DHB because the carbonyl group on the six-membered ring of TNX is converted into the enol structure, showing strong ν_C = C_. In addition, tenoxicam has three different states. The torsion of the C-H bond used in this paper is an important indicator to distinguish between the cocrystal and salt of TNX. In the Raman spectra of TNX-PAS and TNX-DNB cocrystals, TNX is composed of dimers in the zwitterionic form. In the Raman spectra of the TNX-DHB salt, there are two sharp characteristic peaks at 1262 cm ^−1^ and 1299 cm ^−1^, indicating that due to the interaction between DHB and TNX, the C-H structure in the TNX structure is twisted, and the hydrogen on the carboxyl of DHB is transferred to the N of the pyridine ring of TNX. An ionic bond between N-H and C-O was formed, and a hydrogen bond between N-H and C-O was formed. However, there was no such characteristic peak in TNX-PAS and TNX-DNB. Therefore, Raman spectroscopy can effectively distinguish between the TNX eutectic and salt. TNX exists in three different forms, and the reversal of the C-H bond used in this study is an important marker to distinguish the cocrystal from the salt. The two sharp peaks appear at 1262 cm ^−1^ and 1299 cm ^−1^ in the Raman spectra of TNX-DHB, which shows that the C-H structure of TNX was reversed due to the interaction between DHB and TNX. The hydrogen on the carboxyl group of DHB was transferred to the N of the pyridine ring belonging to TNX, which formed ionic bonds between N-H and C-O and hydrogen bonds between N-H and C=O. However, no such peaks existed in TNX-PAS and TNX-DNB. Therefore, Raman spectroscopy is an effective method to distinguish between the cocrystal and salt of TNX.

### 3.6. Differential Scanning Calorimetry (DSC) Analysis

**Figure 6:**
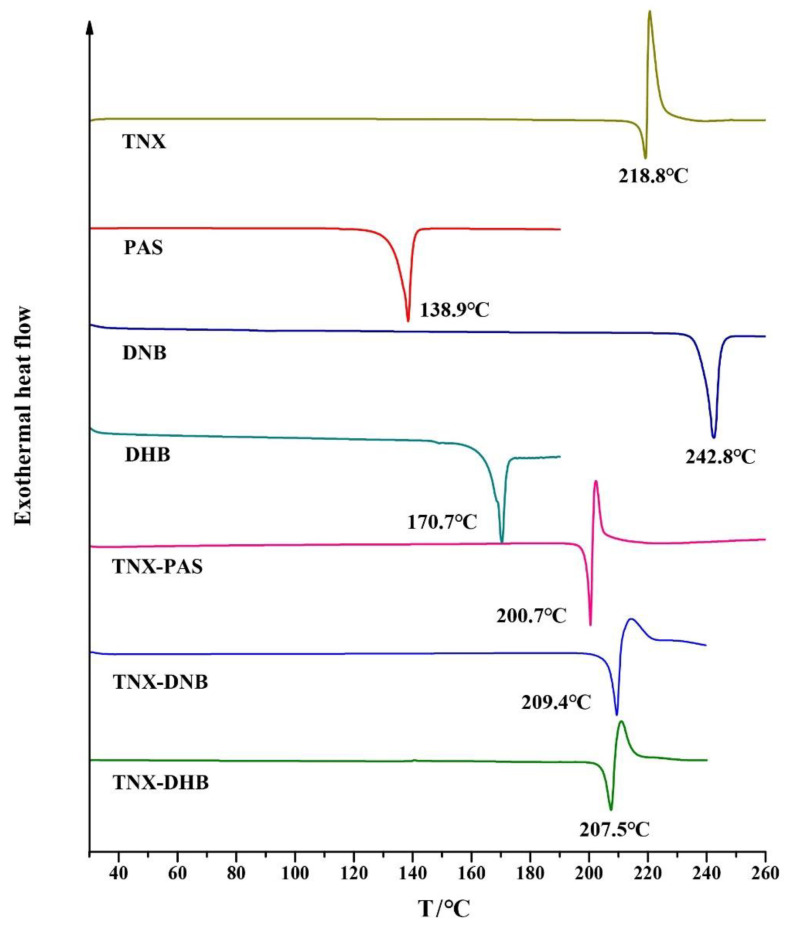
DSC profiles of TNX, CCFs, and the corresponding cocrystals/salt.

**Figure 7:**
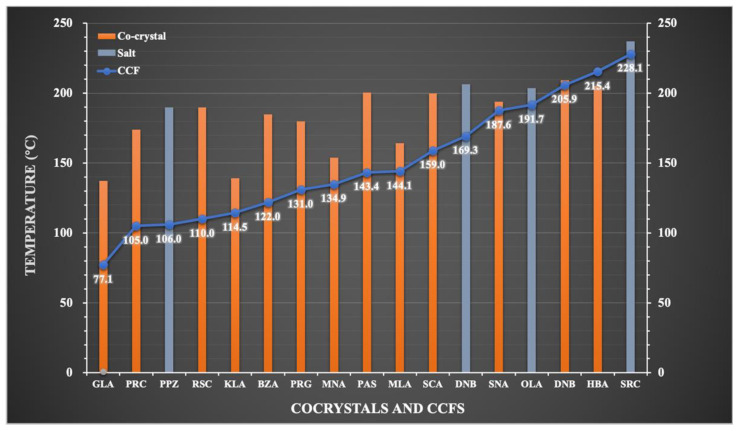
Melting point trend diagram of cocrystal/salt and CCF.

DSC analysis was performed to determine the melting points of the cocrystals/salts and to study their thermal behavior, as shown in Figure 6. TNX-PAS, TNX-DNB, and TNX-DHB showed sharp endothermic peaks at 200.48 °C, 209.26 °C, and 206.38 °C, respectively, which are different from those of API and CCFs, indicating the formation of new phases. The order of peak values from high to low is TNX-DNB > TNX-DHB > TNX-PAS, which is consistent with the order of the melting points of CCF and the crystal density of the cocrystals and salts. It is speculated that lower crystal density results in looser crystal packing and weaker interactions, thus leading to lower melting points [48]. We plotted the melting points of the cocrystals or salts and CCFs listed in Table 1 (Figure 7), and the results show that although the trends of the melting points of cocrystals and salts are not consistent with those of CCFs from low to high, the CCF of the cocrystal with the lowest melting point also had the lowest melting point, and the CCF of the cocrystal with the highest melting point also had the highest melting point. It can be seen that the thermal stability of CCFs has a great influence on the thermal stability of the cocrystal and salt of TNX. In addition, TNX-PAS, TNX-DNB, and TNX-DHB decomposed immediately after melting, similar to the melting behavior of TNX (Figure S1). Although the melting points of the cocrystals and salt were lower than that of TNX, they still showed features of melting decomposition. This indicates that TNX might have decomposed at lower temperatures, but the formation of different crystals delayed the process to varying degrees.

### 3.7. Lattice Energy Analysis

Lattice energy can be used to compare the thermodynamic stability of crystal structures to a certain extent. The higher the value is, the more stable the structure is. In this work, the lattice energies of TNX-PAS, TNX-DNB, and TNX-DHB are −43.25, −48.72, and −125.82, respectively. The enthalpy-of-fusion values of TNX-PAS, TNX-DNB, and TNX-DHB determined by DSC are −498.11 mJ, −965.18 mJ, and −144.31 mJ, respectively. For the two cocrystals, because they all belong to molecular crystals, TNX-DNB is more stable than TNX-PAS due to its higher lattice energy, which is consistent with the DSC analysis results. For the TNX-DHB salt belonging to ionic crystals, its lattice energy is usually larger than that of the molecular crystal, so it is not suitable for direct comparison with the molecular crystal. The enthalpy-of-fusion values of three TNX solid forms also proved this point.

### 3.8. Energy Decomposition Analysis (EDA)

Energy decomposition is an important component of the analytical method of quantum chemistry, and this process can decompose the total interaction energy between molecules into energy terms of physical significance to investigate the nature of the interaction [49,50,51]. Generalized Kohn–Sham EDA (GKS-EDA) decomposes the total interaction energy of the complex (Δ*E*^total^) into five parts, i.e., the electrostatic energy (Δ*E*^ele^), the exchange energy (Δ*E*^ex^), the repulsion energy (Δ*E*^rep^), the polarization energy (Δ*E*^pol^), and the electron correlation energy (Δ*E*^disp^). In this study, GSK-EDA was used to analyze the total interactions of the tetramers (constitutional repeating unit) in the cocrystals and salt (shown in Figure 2) to comprehensively understand the multi-body interaction.

**Figure 8:**
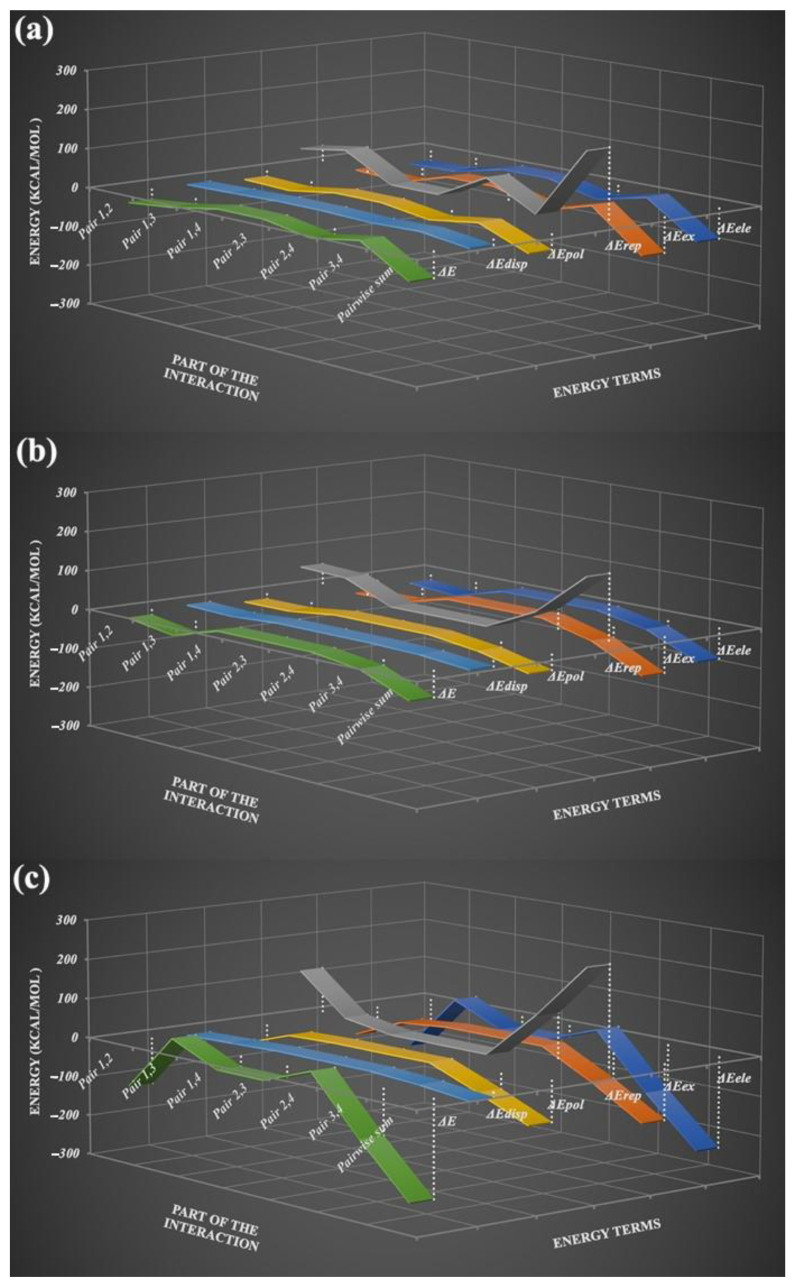
GSK-EDA of the cocrystals and salt of TNX, (**a**) TNX-DNB, (**b**) TNX-PAS, and (**c**) TNX-DHB.

Because the compositions of TNX-PAS, TNX-DNB, and TNX-DHB were different, it was not possible to judge the strength of the intermolecular interactions of different complexes by directly comparing the total interaction energy. However, by using EDA, differences in the components of interactions among these complexes can be revealed. The results of EDA showed that attractive intermolecular interactions of the fragments in the cocrystals and salt were dominated by Δ*E*^ele^ and Δ*E*^ex^. For the two cocrystals, the electrostatic interactions were the manifestation of intermolecular hydrogen bonds, and they were attractive energy. For the salt, the electrostatic interactions were the electrostatic attraction between opposite ions and the electrostatic repulsion between like ions. Δ*E*^ex^ is the energy contribution of orbital relaxation from monomers to the supermolecule, and it can be seen that the exchange energy of the salt is higher than that of the cocrystal because of proton transfer in the salt. Similarly, the proportion of polarization energy in the salt as the attractive energy was larger than that in the cocrystals. The dispersion energy was the minimum, but its effect cannot be ignored. The three-dimensional structures of the crystals are based on the interactions of these kinds of energies. The results of the GSK-EDA of the cocrystals and salt of TNX are plotted as a three-dimensional line chart in Figure 8, and data are listed in Tables S1–S3 in the Supplementary Materials.

### 3.9. Dissolution Measurement Analysis

**Figure 9:**
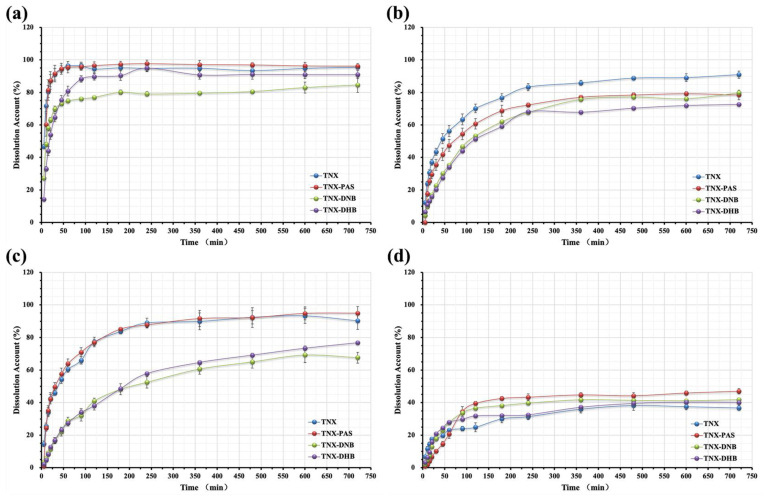
Powder dissolution profiles of the cocrystals and salt of TNX in four solvent systems. (**a**) Hydrochloric buffer (pH 1.2); (**b**) acetate buffer (pH 4.5); (**c**) phosphate buffer (pH 6.8); and (**d**) water (pH 7.0).

Figure 9 shows the results of dissolution measurements of TNX, TNX-PAS, TNX-DNB, and TNX-DHB in four buffers. TNX-PAS had similar solubility features to TNX, and its solubility was slightly higher than that of TNX at pH 1.2 and 6.8, but there were no solubility differences, relatively. The solubility of TNX-DNB was lower than those of TNX, TNXPAS, and TNX-DHB at pH 1.2 and 6.8 and slightly higher than that of TNX-DHB at pH 7.0. TNX-DHB also had similar solubility characteristics to TNX, which was slightly lower. In general, the solubility of the three samples was not improved compared to API, which may be related to the solubility of CCFs.

## 4. Conclusions

In this work, different solid forms that existed in the cocrystals and salt of TNX were carefully investigated, and a simple rule was derived as follows: when the CCF is an acidic compound and the pKa value is less than 2, or the CCF is a basic compound and its pKa value is greater than 9, the salt will be formed, and TNX will exist in enol form I or II, respectively; when the pKa value of the CCF is out the range mentioned above, the cocrystal will be formed, and TNX will exist in the zwitterionic form. According to this rule, two cocrystals and one salt of TNX were synthesized for the first time. In the thermal analysis, we found that the melting point of CCF determined that of the cocrystal or salt, and the thermal stability of TNX was determined. In addition, theoretical calculations, such as lattice energy and energy decomposition, were performed to analyze the intermolecular interactions in the cocrystals and salt, which made the strength of intermolecular forces clearer and the difference between the salt and cocrystal more intuitive. Finally, although neither the cocrystals nor the salt can improve the solubility of TNX, the results suggest that the solubility of the CCF itself plays an important role, and the improvement of solubility after salt formation is not necessarily better than that of API or its cocrystals.

## Figures and Tables

**Table 1:** The melting point, pKa, and ΔpKa of different CCFs and TNX cocrystals.

CCF	Melting Point	Cocrystal Melting Point	pKa ^i^ Value	ΔpKa Value	Conformation of TNX
Glycolic acid (GLA)	77.1	137.2	3.74	0.76	Unknown
4-Hydroxybenzoic acid (HBA)	215.4	205.2	4.57	0.07	Unknown
*α*-Ketoglutaric acid (KLA)	114.5	139.3	2.38	2.12	Unknown
Succinic acid (SNA)	187.6	194	4.24	0.26	Unknown
Maleic acid (MLA)	144.1	164.2	2.39	2.11	Unknown
Malonic acid (MNA)	134.9	154	2.92	2.11	Unknown
Oxalic acid (OLA)	191.7	203.6	1.38	1.58	Unknown
Saccharin (SCR)	228.1	237.1	1.60	3.12	Unknown
Benzoic acid (BZA)	122	185	4.20	1.49	A
Salicylic acid (SCA)	159	200	3.01	0.30	A
Pyrocatechol (PRC)	105	174	9.50	5.00	A
Resorcinol (RSC)	110	190	9.45	4.95	A
Pyrogallol (PRG)	131	180	9.28	4.78	A
Piperazine (PPZ)	106	190	9.55	5.05	B
Hydrochloric acid (HCA)	—	198	−8	12.50	B
Methanesulfonic acid (MSA)	—	209	1.75	2.75	B
*p*-Aminosalicylic acid (PAS)	143.4	200.5	3.58	0.92	A
3,5-Dinitrobenzoic acid (DNB)	205.9	209.3	2.77	1.73	A
2,6-Dihydroxybenzoic acid (DHB)	169.3	206.4	1.30	3.20	B

^i^: The melting points and pKa values in the table were obtained from SciFinder. “A” represents the zwitterionic form, and “B” represents enol form I or enol form II. —: The coformer compound is liquid.

**Table 2:** Crystal Cell Parameters and Structure Refinement of the Cocrystals or Salt of TNX.

	TNX-PAS	TNX-DNB	TNX-DHB
Formula	C_13_H_11_N_3_O_4_S_2_∙C_7_H_7_NO_3_	C_13_H_11_N_3_O_4_S_2_∙C_7_H_4_N_2_O_6_	C_13_H_11_N_3_O_4_S_2_∙C_7_H_6_O_4_
Description	flake	flake	flake
Crystal system	monoclinic	triclinic	monoclinic
Space group	*P* 2_1_	*P* $\bar{1}$	*C* 2/c
Unit cell parameters (Å, °)	9.220 (1)	9.578 (1)	21.516 (1)
	20.215 (1)	10.721 (1)	7.731 (1)
	11.617 (1)	11.617 (1)	26.440 (1)
	90	93.248 (3)	90
	97.98 (1)	107.587 (3)	104.070 (10)
	90	99.406 (2)	90
Volume (Å^3^)	2144.65 (2)	1114.75 (6)	4266.37 (11)
Z	4	2	2
Density (g/cm^3^)	1.519	1.637	1.530
Theta range for data collection	3.84~72.14	4.01~71.55	3.42~72.16
Independent reflections	7115	4187	4166
Reflections with I > 2σ(I)	6192	3814	3694
R (I > 2σI)	R = 0.052	R = 0.0404	R = 0.0571
	wR_2_ = 0.147	wR_2_ = 0.1146	wR_2_ = 0.1599
Goodness-of-fit on F^2^	1.066	1.086	1.025
Completeness	99.3	97.6	99.7
Deposition number	2174287	2174286	2174285

**Table 3:** Parameters (Å, °) of Main Hydrogen Bonds for Cocrystals and Salt of TNX.

Cocrystal/Salt	D–H…A	D…A (Å)	∠DHA (°)
TNX-PAS	N_2_-H_2_…O_1_	2.607	140.28
	N_3_-H_3_…O_4_	2.651	131.79
	N_3_-H_3_…O_8_ ^a^	2.894	139.58
	N_1A_-H_1AB_…O_3_	3.017	152.46
	O_1A_-H_1A_…O_1_ ^b^	2.572	160.00
	O_3A_-H_3AA_…O_2A_	2.624	137.63
TNX-DNB	N_2_-H_2_…O_1_	2.637	138.40
	N_3_-H_3_…O_4_ (intra-)	2.585	133.03
	N_3_-H_3_…O_4_ ^c^ (inter-)	2.991	135.57
	O_2D_-H_2D_…O_1_	2.572	166.51
TNX-DHB	N_2_-H_2_…O_5_	3.018	168.02
	N_3_-H_3A_…O_6_	2.593	157.48
	O_1_-H_1_…O_4_ (intra-)	2.559	142.82
	O_1_-H_1_…O_4_ ^d^ (inter-)	2.886	122.41
	O_7_-H_7_…O_6_	2.529	146.91
	O_8_-H_8_…O_5_	2.568	147.81

Symmetry Codes: ^a^ x + 1, y, z; ^b^ x + 1, y, z − 1; ^c^ −x + 2, −y + 1, −z + 1; ^d^ −x + 1, y, −z + 1/2.

## Data Availability

CCDC 2174285, 2174286, and 2174287 contain the supplementary crystallographic data for this paper. These data can be obtained free of charge via www.ccdc.cam.ac.uk/data_request/cif (accessed on 1 September 2022) or by emailing data_request@ccdc.cam.ac.uk or by contacting The Cambridge Crystallographic Data Centre, 12 Union Road, Cambridge, CB2 1EZ, UK; Fax: +441223-336033.

## Supplementary Materials

The following supporting information can be downloaded at: https://www.mdpi.com/article/10.3390/pharmaceutics14091968/s1. Table S1: GSK-EDA of the TNX-PAS cocrystal; Table S2: GSK-EDA of the TNX-DNB cocrystal; Table S3: GSK-EDA of the TNX-DHB salt; Figure S1: DSC and TG profiles of TNX, CCFs, and the corresponding cocrystals/salt.
