# Supplementary material for: Insight into the Formation of Cocrystal and Salt of Tenoxicam from the Isomer and Conformation
Source: Pharmaceutics. 2022 Sep 19;14(9):1968. doi: 10.3390/pharmaceutics14091968 (PMC9504695; doi:10.3390/pharmaceutics14091968)
Supplement: Supplementary file 1 [file pharmaceutics-14-01968-s001.zip › Supplementary materials.pdf]

Table S1. GSK-EDA of the cocrystal TNX-PAS (kcal/mol).

Pair	ΔE^{ele}	ΔE^{ex}	ΔE^{rep}	ΔE^{pol}	ΔE^{disp}	ΔE
Pair 1,2	-24.20	-31.69	57.78	-14.89	-3.11	-16.11
Pair 1,3	-33.94	-30.49	51.98	-14.57	-7.56	-34.58
Pair 1,4	0.58	0.00	0.00	0.13	-0.14	0.57
Pair 2,3	0.59	0.00	0.00	0.13	-0.14	0.58
Pair 2,4	-0.02	0.00	0.00	-0.01	0.01	-0.02
Pair 3,4	-25.02	-33.25	60.9	-15.81	-3.04	-16.23
Pairwise sum	-82.01	-95.43	170.66	-45.02	-13.98	-65.79
Total interaction	-81.78	-95.33	170.48	-42.28	-14.36	**-63.26**
Many-body effects	0.23	0.10	-0.18	2.74	-0.38	2.53

Table S2. GSK-EDA of the cocrystal TNX-DNB (kcal/mol).

Pair	ΔE^{ele}	ΔE^{ex}	ΔE^{rep}	ΔE^{pol}	ΔE^{disp}	ΔE
Pair 1,2	-31.54	-28.17	47.63	-13.64	-7.18	-32.89
Pair 1,3	-28.48	-36.29	66.31	-18.12	-3.97	-20.54
Pair 1,4	0.68	0.00	0.00	0.09	-0.11	0.67
Pair 2,3	0.68	0.00	0.00	0.09	-0.11	0.66
Pair 2,4	-28.49	-36.29	66.33	-18.14	-3.96	-20.57
Pair 3,4	-0.01	0.00	0.00	0.00	0.00	-0.01
Pairwise sum	-87.16	-100.75	180.27	-49.72	-15.33	-72.68
Total interaction	-86.9	-100.65	180.11	-46.46	-15.73	**-69.64**
Many-body effects	0.26	0.10	-0.16	3.26	-0.40	3.04

Table S3. GSK-EDA of the salt TNX-DHB (kcal/mol).

Pair	ΔE^{ele}	ΔE^{ex}	ΔE^{rep}	ΔE^{pol}	ΔE^{disp}	ΔE
Pair 1,2	-115.84	-65.95	121	-49.03	-8.45	-118.26
Pair 1,3	28.23	-11.53	19.83	-4.94	-4.44	27.16
Pair 1,4	-26.4	0	0	-0.47	0.1	-26.78
Pair 2,3	-26.41	0	0	-0.48	0.1	-26.78
Pair 2,4	20.75	0	0	0.47	-0.53	20.69
Pair 3,4	-115.8	-65.87	120.84	-48.98	-8.45	-118.27
Pairwise sum	-235.47	-143.35	261.67	-103.43	-21.67	-242.24
Total interaction	-235.71	-143.3	261.72	-102.64	-22.42	**-242.34**
Many-body effects	-0.24	0.05	0.05	0.79	-0.75	-0.1

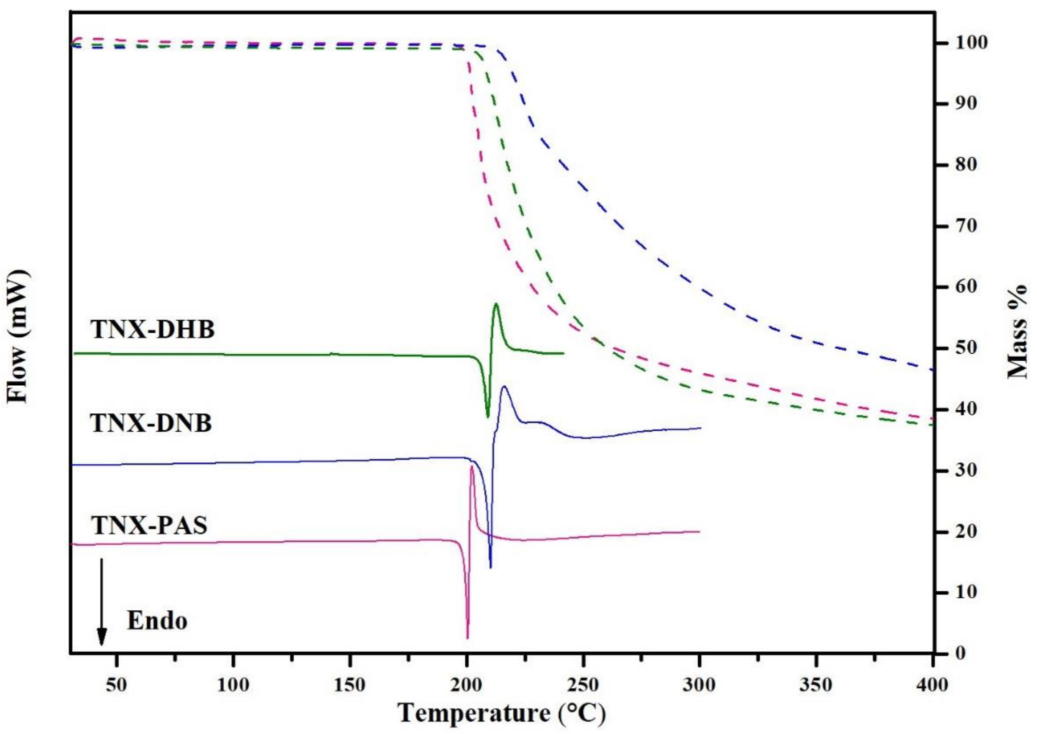

Figure S1. DSC and TG profiles of TNX, CCFs and the corresponding cocrystals/salt.
